# miR-31-mediated local translation at the mitotic spindle is important for early development

**DOI:** 10.21203/rs.3.rs-3044775/v1

**Published:** 2023-06-14

**Authors:** Carolyn M. Remsburg, Kalin Konrad, Nadezda Stepicheva, Michael Testa, Kelvin Lee, Leila Choe, Shawn Polson, Jaysheel Bhavsar, Hongzhan Huang, Jia L. Song

**Affiliations:** 1 Department of Biological Sciences, University of Delaware, Newark, DE 19716; 2 Department of Neurology, Columbia University, New York, NY 10032; 3 Department of Ophthalmology, University of Pittsburgh, Pittsburgh, PA 15224; 4 Department of Chemical and Biomolecular Engineering, University of Delaware, Newark, DE 19716; 4 National Institute for Innovation in Manufacturing Biopharmaceuticals, Newark, DE 19716; 5 Department of Computer and Informational Sciences; Plant & Soil Sciences; Biological Sciences, CBCB Bioinformatics Core Facility; Bioinformatics, Healthcare Informatics, and Data Science Network of Delaware, University of Delaware, Newark, DE 19716; 6 Department of Computer and Informational Sciences, University of Delaware, DE 19716; 7 Department of Computer and Informational Sciences, University of Delaware, DE 19716

## Abstract

miR-31 is a highly conserved microRNA that plays critical roles in cell proliferation, migration, and differentiation. We discovered miR-31 and some of its validated targets are enriched on the mitotic spindle of the dividing sea urchin embryo and mammalian cells. Using the sea urchin embryo, we found that miR-31 inhibition led to developmental delay correlated with increased cytoskeleton and chromosomal defects. We identified miR-31 to directly suppress several actin remodeling transcripts, *β-actin, Gelsolin*, *Rab35* and *Fascin*, which were localized to the mitotic spindle. miR-31 inhibition leads to increased newly translated Fascin at the spindles. Forced ectopic localization of *Fascin* transcripts to the cell membrane and translation led to significant developmental and chromosomal segregation defects, leading to our hypothesis that miR-31 regulates local translation at the mitotic spindle to ensure proper cell division. Furthermore, miR-31-mediated post-transcriptional regulation at the mitotic spindle may be an evolutionarily conserved regulatory paradigm of mitosis.

## Introduction

Mitosis is a fundamental process that results in the faithful segregation of chromosomes to each resulting daughter cell^1^ . Misregulation of mitosis can lead to cell death, arrest or DNA damage^2,3^. Faithful segregation of chromosomes into daughter cells is mediated by the mitotic spindle which is composed primarily of microtubules^4^ and associated proteins. The mitotic spindle is regulated by a complex set of RNAs, microtubule motors, polymerizing factors, as well as by actin^5^. To date, post-transcriptional regulation mediated by microRNAs at the mitotic spindle has not been examined.

The long-held belief has been that translation is generally repressed during mitosis^6^. However, these studies were often performed using cell-cycle synchronization techniques that rely on microtubule disruption or inhibition of DNA synthesis, which stress the cell and lead to global translational repression^6^. In recent studies, it has been shown that the magnitude of this repression is considerably less than previously thought. For example, using CDK inhibition leads to only a 35% reduction in translation, while temperature-sensitive mutants of *cdc-10*, *cdc-25* and *nda3* and fluorescence activated cell sorting on non-synchronized cells reveal no significant cell-cycle dependent differences in translation^6,7^. Additional data suggest that mitosis-related proteins, such as cohesins, cyclins, and mitotic spindle components are more likely to be translated during mitosis^8^. These new data indicate that translation is not globally inhibited during mitosis, but rather, specific proteins are being translated during this time.

Evidence of local translation during mitosis has also emerged, as translational initiation and elongation factors, ribosomal proteins, and various RNA species have been observed on the mitotic spindle^9–12^. *Cyclin B* translated at the mitotic spindle is important for the progression of mitosis in frog embryos^13^. The RNA-binding protein Vasa regulates translation at the mitotic spindle in order to mediate asymmetric protein accumulation after unequal cell division in sea urchin embryos, and *Vasa* itself appears to be translated on the mitotic spindle^12^. Biochemical analyses have revealed that a complex pool of mRNAs, lncRNAs and miRNAs associate with the mitotic spindle^10,14^. Many of these spindle-associated mRNAs encode proteins that regulate various aspects of mitosis^14^. Furthermore, we have recently identified several mRNAs encoding proteins that regulate mitosis localize to the mitotic spindle^15^. Disruption of the localization of one of these transcripts, *AuroraB,* results in early embryonic developmental delay and lethality^15^. These studies and others strongly suggest that subcellular localization of transcripts and their translation at the mitotic spindle may play an important role in the progression of mitosis and thus early development.

Post-transcriptional regulation by microRNAs occurs in the majority of protein-coding genes, generally negatively regulating translation of target genes^16^, and therefore, may be a mechanism to regulate local translation at the mitotic spindle. In this study, we use the purple sea urchin embryo to identify the regulatory role of miR-31 during mitosis, as miR-31 is one of the most expressed microRNAs that is essential in rescuing sea urchin embryonic lethality induced by *Drosha* and/or *Dicer* knockdown (KD)^17^. Our current study reveals that miR-31 is essential for proper early embryonic development, with inhibition of miR-31 resulting in developmental delay and lethality of cleavage stage embryos which exhibit chromosomal defects and increased tubulin and F-actin. The early cleavage stage of development in metazoans is characterized by a series of rapid cell divisions^18^, during which efficient and rapid protein regulation is important. In examining the role miR-31 plays in development^19,20^, we discovered that miR-31 has a cell-cycle dependent subcellular localization to the perinuclear region and the mitotic spindle. Using high-throughput approaches, we identified and validated several miR-31 targets, including *β-actin*, *Gelsolin*, *Rab35*, and *Fascin*, some of which have evolutionarily conserved localization to the mitotic spindle in sea urchin embryos and mammalian cells. Our results indicate that inhibition of miR-31 leads to increased *de novo* Fascin protein synthesis especially at the mitotic spindle. Forced ectopic translation of *Fascin* at the cell membrane results in developmental delay, leading to the hypothesis that miR-31 mediates local translation of cytoskeletal modulating transcripts such as *Fascin* and *Rab35* at the mitotic spindle to fine-tune mitosis. Fascin cross-links F-actin into linear bundles, and interacts with and promotes microtubule polymerization^21–23^; Rab35 is a small GTPase that directly interacts with Fascin and regulates actin polymerization^24,25^. We propose that regulated local translation of Fascin and Rab35 at the mitotic spindle may allow rapid polymerization of the cytoskeleton that is needed to mediate the timely segregation of chromosomes. This is the first study that demonstrates that microRNA mediated post-transcriptional regulation at the mitotic spindle is important for faithful chromosomal segregation and early development. Importantly, this regulation of mitosis may be evolutionarily conserved.

## Results

### miR-31 has a dynamic localization that correlates to the cell cycle.

**Figure 1:**
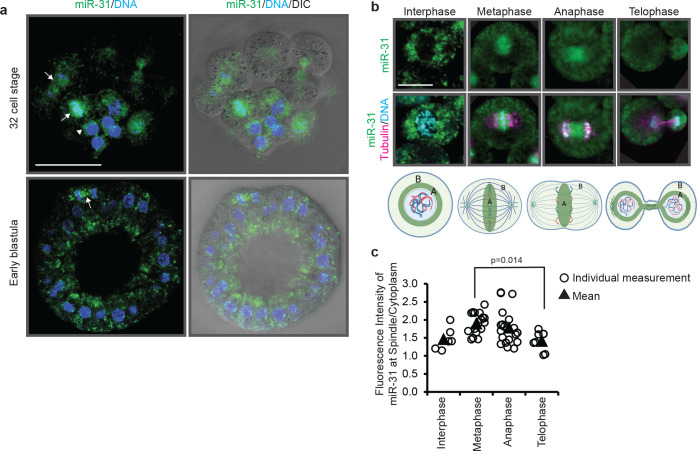
miR-31 localizes to the mitotic spindles in a cell-cycle dependent manner. (a) miR-31 is located in at the mitotic spindle in dividing cells (white arrows), and perinuclearly in cells in interphase (arrowhead). 16–32 cell stage embryos were subjected to miR-31 FISH (green) and counterstained with Hoechst dye to detect DNA (blue). Scale bar = 50μm. (b) Single blastomeres of a 16–32 cell stage embryo are depicted. Images were obtained by miR-31 FISH (green) followed by immunolabeling with β-tubulin antibody (magenta) and counterstained with DAPI to detect DNA (blue). Scale bar = 10μm. 3 biological replicates. (c) ImageJ was used to quantify the enrichment of miR-31 during different cell cycle phases. N=6 interphase blastomeres, 16 metaphase blastomeres, 20 anaphase blastomeres, 8 telophase blastomeres. p-value was obtained using an ANOVA with a Tukey-Kramer post-hoc test.

In examining the role of miR-31 in development, we discovered that miR-31 has a cell cycle-dependent distribution. miR-31 is enriched on the mitotic spindle in dividing cells and in the perinuclear region of non-dividing cells of sea urchin embryos (Fig. 1a). The association of miR-31 with the mitotic spindle continues to at least the early blastula stage. Based on the structure of the chromosomes and microtubules, we analyzed miR-31’s localization enrichment throughout stages of the cell cycle (Fig. 1b). During interphase, miR-31 is enriched in the perinuclear region; as the blastomere progresses from metaphase to anaphase, miR-31 is enriched at the spindle midzone. During telophase, a significant decrease in enrichment at the mitotic spindle is observed, compared to its enrichment at the metaphase (Fig. 1c).

### miR-31 inhibited embryos are developmentally delayed, exhibit chromosomal segregation defects, and have increased microtubules and F-actin.

To test the function of miR-31, we injected a miR-31 locked-nucleic acid (LNA) inhibitor into newly fertilized eggs, using a miR-124 LNA inhibitor as a control for LNA toxicity. miR-124 is not expressed until 12 hours post fertilization (hpf)^26^ and embryos injected with miR-124 LNA inhibitor exhibit normal early cleavage stage development, similar to control embryos injected with a dextran (Fig. S1). Using fluorescence *in situ* hybridization (FISH) to assess the efficacy of the miR-31 inhibitor, we observe a significant decrease in detectable miR-31 in miR-31 inhibitor-injected embryos, indicating that the majority of endogenous miR-31 is likely to be bound to the miR-31 inhibitor and not available for miR-31 RNA probe binding (Fig. S2).

**Figure 2:**
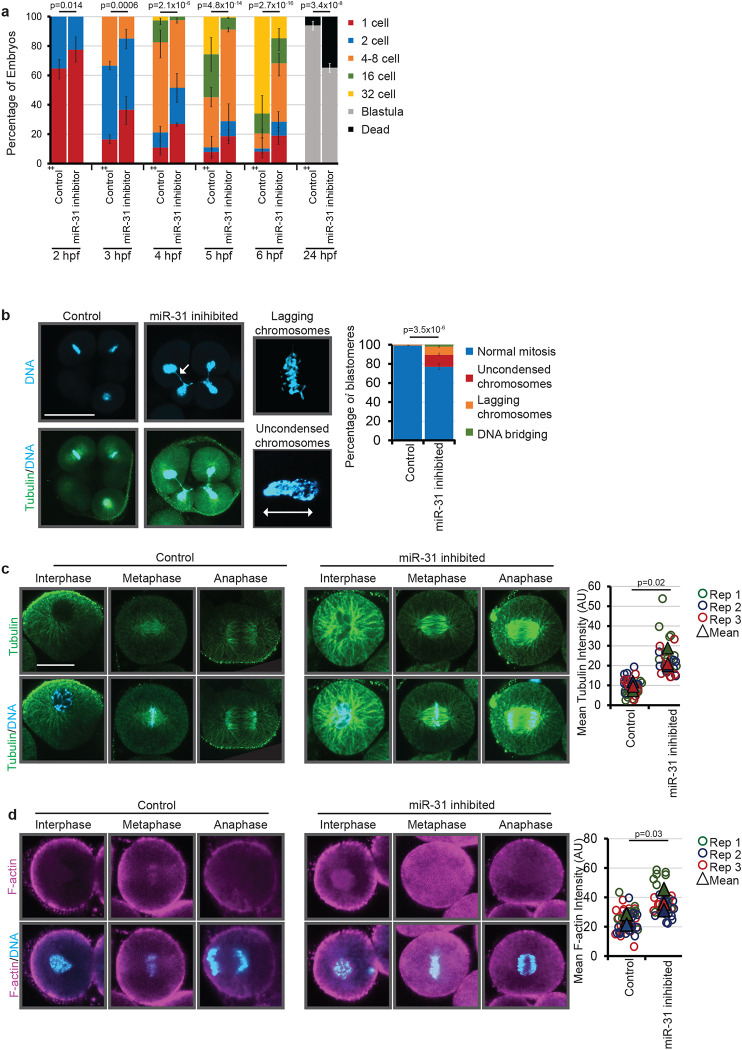
miR-31 inhibition results in embryonic lethality. (a) Zygotes were injected with miR-31 inhibitor or control miR-124 inhibitor. The number of embryos in each developmental stage was recorded every hour for 6 hours post-fertilization (hpf), then again at 24 hpf. p-values were obtained using Cochran-Mantel-Haenszel test. N=212 Control miR-124 inhibitor injected embryos,128 miR-31 inhibited embryos, 3 biological replicates. (b) miR-31 inhibited embryos exhibit chromosomal bridging, lagging chromosomes (arrows), and uncondensed chromosomes that may result in aneuploidy. Embryos were injected with miR-31 LNA inhibitor or control miR-124 LNA inhibitor and immunolabeled for tubulin in green and counterstained DNA with DAPI in blue. Scale bar = 50μm. Blastomeres undergoing mitosis were scored for chromosomal abnormalities as indicated. p-values were obtained using Cochran-Mantel-Haenszel test. N=138 Control miR-124 inhibitor-injected blastomeres, N=139 miR-31 inhibitor-injected embryos. 3 biological replicates (c) miR-31 inhibition results in increased interpolar and astral microtubules. Embryos were injected with miR-31 LNA inhibitor or control miR-124 LNA inhibitor and immunolabeled for tubulin in green and counterstained DNA with DAPI (blue). ImageJ was used to measure the fluorescence intensity of individual blastomeres during anaphase. N=37 control blastomeres, 30 miR-31 inhibited blastomeres. p-values were obtained using Student’s t-test. (d) Control embryos exhibit enriched filamentous actin (F-actin) at the cell cortex and surrounding the chromosomes. miR-31 inhibited embryos exhibit an increase in F-actin, particularly surrounding the chromosomes. Zygotes were injected with miR-31 LNA inhibitor and labeled with Alexa647-phalloidin (magenta) and counterstained with DAPI (blue). ImageJ was used to measure the fluorescence intensity of single blastomeres. N= 49 control blastomeres, 40 miR-31 inhibited blastomeres. p-values were obtained using Student’s t-test.

We observe that inhibition of miR-31 results in a significant delay or arrest in embryonic development compared to the control miR-124 inhibitor-injected embryos (Fig. 2). As early as 2 hpf, we observed significant differences between control and miR-31 inhibitor-injected embryos. This significant difference in development continues to 6 hpf, where 66% and 14% of control and miR-31 inhibitor-injected embryos develop to the 32-cell stage, respectively (Fig. 2a). This difference in development persists to the blastula stage, as 90% and 70% of the control and miR-31 inhibitor-injected embryos survived to the blastula stage, respectively (Fig. 2a).

As miR-31 inhibition leads to early developmental delay and/or arrest at a time when the embryos rapidly divide, we investigated chromosomal integrity in these early cleavage stage embryos. In interphase blastomeres, we observed no significant differences in their chromosomal structure (Fig. 2b). However, in dividing blastomeres, we observed that approximately 25% of blastomeres exhibit chromosomal defects, including uncondensed chromosomes, lagging chromosomes, or DNA bridging (Fig. 2b). These results suggest that miR-31 inhibition leads to chromosomal segregation defects.

As proper chromosomal segregation is dependent upon formation of the mitotic spindle, we examined the structure of the cytoskeleton. miR-31 inhibition results in significantly increased microtubules, including interpolar and kinetochore microtubules during anaphase (Fig. 2c), as well as significantly increased filamentous actin (F-actin) in miR-31 inhibited blastomeres in anaphase compared to the control-injected embryos (Fig. 2d).

### High throughput identification of miR-31 targets reveal targets that encode regulators of cytoskeletal dynamics.

**Figure 3:**
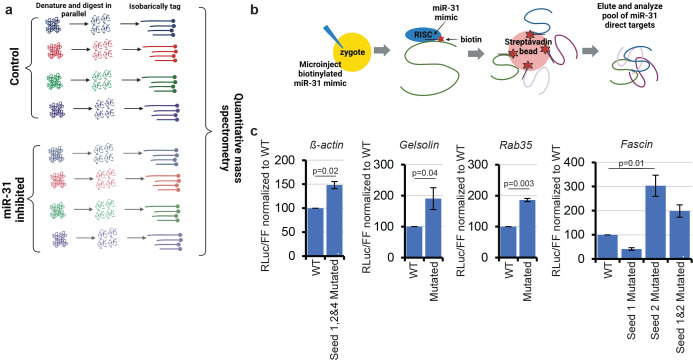
Identification and validation of miR-31 targets. (a) Embryos were injected with a biotinylated miR-31 mimic, which was incorporated into the RNA Induced Silencing Complex (RISC). Embryo lysate was then incubated with streptavidin beads. Transcripts bound to the biotinylated miR-31 were eluted and subjected to RNA-Seq. (b) Embryos were injected with scrambled LNA negative control or miR-31 LNA inhibitor. The proteomes were collected and isobarically tagged and submitted for quantitative mass spectrometry. (c) Potential targets were cloned downstream of *Renilla* luciferase (RLuc). Mutagenesis was performed on the predicted miR-31 seed sequences. After the constructs were *in vitro* transcribed and microinjected into zygotes, along with firefly luciferase (FF) as a normalization control, the embryos were collected and lysed 24hpf and assayed using the dual luciferase assay. p-values were obtained using a two-tailed Student’s t-test for *β-actin, Rab35* and *Fascin*, and a one-tailed Student’s t-test for *Gelsolin*; 3 biological replicates, error bars represent SEM.

To determine potential miR-31 targets, we took two different high throughput approaches (Fig. 3). Three biological replicates of zygotes were injected either with a scramble control or miR-31 inhibitor and subjected to 8-plex isobaric tagging methods for the quantitation of proteins (Fig. 3a, Table S1). Proteins that had increased levels upon miR-31 inhibition compared to the control were further analyzed bioinformatically. The current microRNA prediction programs scan 3’UTRs for short motifs that are complementary to the microRNA seed sequence in conjunction with evidence of conservation across phylogeny. As this conservation information is not currently available for the sea urchin genome, we took 1.5 Kb of their 3’UTRs and bioinformatically searched for the complement of the miR-31 seed sequences^27,28^. The second approach was to inject biotinylated miR-31 mimic into the zygotes, in which the miR-31 would get incorporated into the RNA-Induced Silencing Complex and bind to endogenous miR-31 targets (Fig. 3b, Table S2). Embryonic lysates were passed over a streptavidin bead-coated column, and the bound RNA transcripts were eluted and identified using RNA sequencing.

From both approaches, we identified a list of potential miR-31 targets. Using a dual luciferase assay and site-directed mutagenesis, we validated a select number of potential targets. Since we identified that miR-31 inhibition leads to cytoskeletal defects (Fig. 2c,d), we focused on transcripts that encode proteins that regulate cytoskeletal dynamics. We determined that miR-31 directly suppresses *β-actin, Gelsolin*, *Rab35* and *Fascin* (Fig. 3c). Of note is that *β-actin* was the only target that was identified from both approaches.

**Figure 4:**
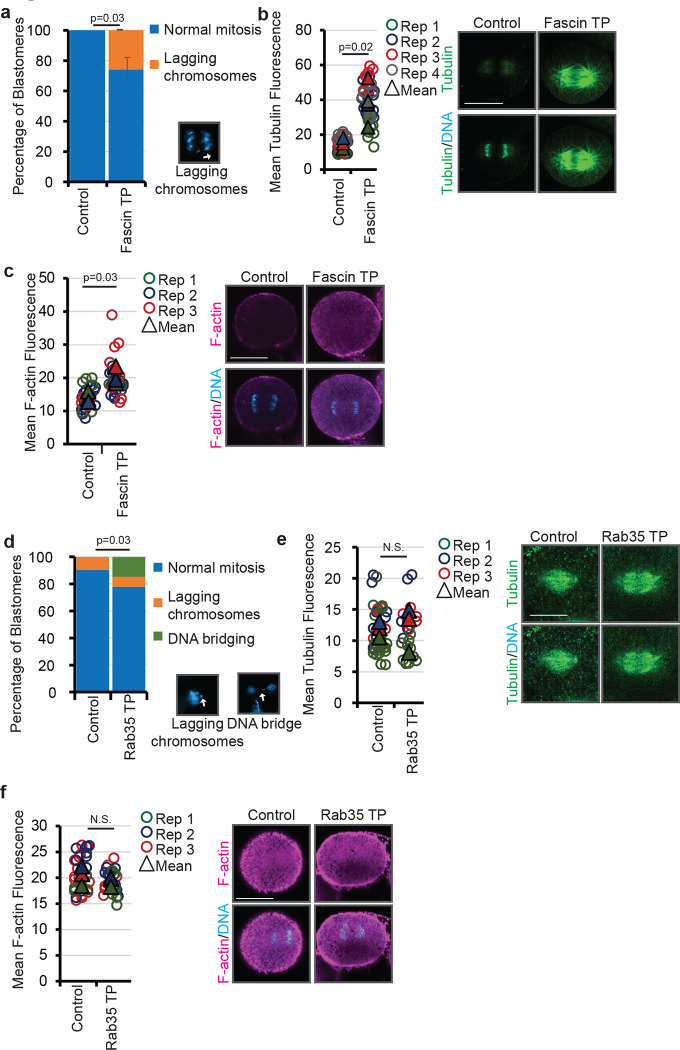
Removing miR-31’s suppression of Fascin leads to chromosomal defects and increased cytoskeletal proteins. (a) Blastomeres undergoing mitosis were scored for chromosomal abnormalities as indicated. Fascin TP-injected embryos exhibit lagging chromosomes that may result in aneuploidy. p-value was obtained using Cochran-Mantel-Haenszel test. N=30 Control TP-injected blastomeres, N=36 Fascin TP-injected embryos. 3 biological replicates (b) Embryos were injected with Fascin TP or control TP and immunolabeled for tubulin in green and counterstained DNA with DAPI (blue). ImageJ was used to measure the fluorescence intensity of individual blastomeres during anaphase. N=30 Control blastomeres, 36 Fascin TP blastomeres. p-value was obtained using Student’s t-test. Scale bar = 50μm (c) Zygotes were injected with control or Fascin TP and labeled with Alexa647-phalloidin (magenta) and counterstained with DAPI (blue). ImageJ was used to measure the fluorescence intensity of single blastomeres. N=26 Control blastomeres, 29 Fascin TP blastomeres. p-value was obtained using Student’s t-test. (d) Blastomeres undergoing mitosis were scored for chromosomal abnormalities as indicated. Rab35 TP-injected embryos exhibit DNA bridges and lagging chromosomes that may result in aneuploidy. p-value was obtained using Fisher’s exact test. N=105 Control TP-injected blastomeres, N=68 Rab35 TP-injected blastomeres. (e) Embryos were injected with control or Rab35 TP and immunolabeled for tubulin in green and counterstained DNA with DAPI (blue). Rab35 TP-injected embryos exhibit no change in tubulin. ImageJ was used to measure the fluorescence intensity of individual blastomeres during anaphase. N.S.= p>0.05 using Student’s t-test. Scale bar = 50μm. (f) Zygotes were injected with control or Rab35 TP and labeled with Alexa647-phalloidin (magenta) and counterstained with DAPI (blue). Rab35 TP-injected embryos exhibit no change in F-actin. ImageJ was used to measure the fluorescence intensity of single blastomeres. N.S.= p>0.05 using Student’s t-test.

To examine if the increase in cytoskeletal proteins induced by miR-31 inhibition may be a result of its suppression of *Fascin* and/or *Rab35*, we injected *Fascin* and *Rab35* target protectors (TPs) into zygotes. TPs are morpholino anti-sense oligonucleotides (MASOs) that we designed to block the binding of miR-31 to the 3’UTR of *Fascin* and *Rab35*. By including nucleotides flanking the validated miR-31 binding site, the TPs bind specifically to the *Fascin* and *Rab35* 3’UTR. *Fascin* TP-injected embryos and *Rab35* TP-injected embryos exhibit more chromosomal defects compared to control embryos (Fig. 4a,d), similar to what we observe in miR-31-inhibited embryos (Fig. 2b). Additionally, Fascin TP-injected embryos exhibit a significant increase in both tubulin and F-actin compared to control embryos (Fig. 4b,c), similar to what was observed in miR-31-inhibited embryos (Fig. 2c,d). However, Rab35 TP-injected embryos exhibit no change in tubulin or F-actin levels compared to control embryos (Fig. 4e,f).

### Spatial distribution of miR-31 and its targets on the mitotic spindle is evolutionarily conserved between sea urchin embryos and mammalian cells.

**Figure 5:**
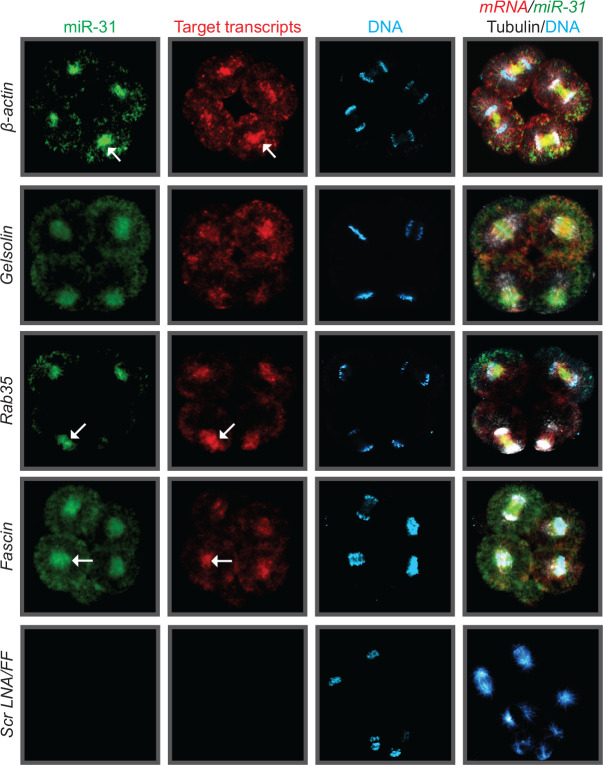
miR-31 and its target transcripts co-localize to the mitotic spindles. 16–32 cell stage embryos were subjected to double FISH (green for miR-31 and red for target transcript) followed by immunolabeling with β-tubulin antibody (white) and counterstained DNA with DAPI (blue). Negative controls are a scrambled LNA and a DNP-labeled FF RNA probe. Scale bar = 50μm.

As microRNAs must bind to their target RNAs to mediate post-transcriptional regulation^16^, we examined where and when miR-31 interacts with its targets. We observed that *β-actin, Gelsolin, Rab35* and *Fascin*, co-localize with miR-31 on the mitotic spindle during mitosis (Fig. 5). During interphase and prophase, miR-31 and its targets localize to the perinuclear region (Figs. 1c,S3). During metaphase and anaphase, miR-31 and its targets localize to the midzone of the mitotic spindle (Figs. 1a,c and 4). During telophase, miR-31’s enrichment to the mitotic spindle is significantly decreased in comparison to metaphase (Fig. 1c).

**Figure 6:**
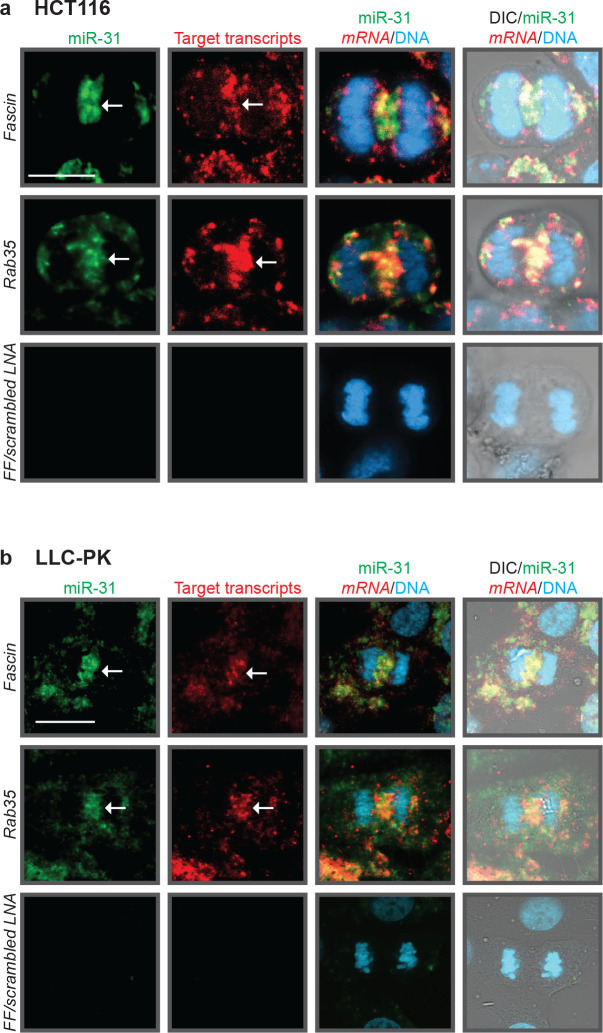
Co-localization of miR-31 and *Fascin* at the mitotic spindle is conserved in mammalian cells and human colon cancer cells. (a) miR-31 and *Fascin* RNA localize between dividing human colon cancer cells (HCT116) (arrow). A scrambled miR LNA probe and FF probe are used as negative controls. 3 biological replicates. (b) miR-31 and *Fascin* RNA localize between dividing pig kidney epithelial cells (LLC-PK) (arrow). A scrambled miR LNA probe and Firefly probe are used as a negative control.3 biological replicates. Scale bar = 10μm.)

To determine if this localization is evolutionarily conserved, we examined the spatial localization of miR-31 and its targets *Fascin* and *Rab35* in HCT116 (human colon cancer cells), which is known to have upregulated miR-31^29^. We observed miR-31 and its targets localize to the spindle midzone in anaphase (Fig. 6a). We also observed a similar co-localization of miR-31 and its targets *Fascin* and *Rab35* during anaphase in a pig kidney cell line, LLC-PK, as these have previously been used to observe RNA localization during mitosis^15^ (Fig. 6b). These results indicate that the co-localization of miR-31, *Fascin*, and *Rab35* is evolutionarily conserved between the sea urchin embryo and mammalian cells.

### *De novo* translation occurs at the mitotic spindle during mitosis.

**Figure 7:**
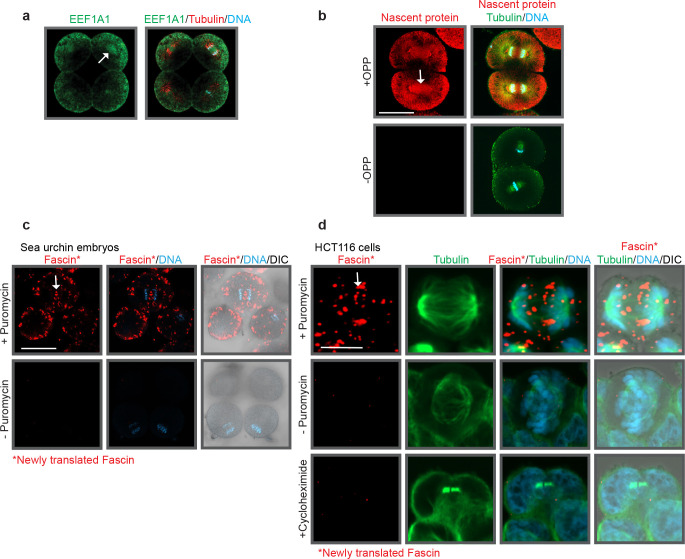
Translational components and newly translated proteins are localized to the mitotic spindle. (a) EEF1A1, a peptide elongating factor, is observed at the mitotic spindle. Embryos were immunolabeled with an EE1A1 (green) and a tubulin antibody (red), then counterstained DNA with DAPI (blue). Scale bar = 50μm. (b) Nascent protein is observed at the mitotic spindle. Embryos were incubated with o-prorargyl puromycin (OPP), a tRNA analogue which was then covalently linked to Alexa 594 fluorophore. Embryos were then immunolabeled with a tubulin antibody (green) and counterstained with DAPI (DNA). Scale bar = 50μm (c) 16–32 cell stage embryos were treated with the DuoLink PuroPLA assay with newly translated Fascin in red, then counterstained with DAPI (DNA in blue). Newly translated Fascin (red) is observed at the cell cortex and mitotic spindle in early cleavage stage blastomeres. Scale bar = 50μm (d) HCT116 cells were treated with the DuoLink PuroPLA assay with newly translated Fascin in red, then immunolabeled with an Alexa-488-conjugated tubulin antibody in green and counterstained with DAPI (DNA blue). Newly translated Fascin is observed at the mitotic spindle in HCT116 cells. Scale bar = 5 μm.

Our results indicate that miR-31 and its target RNAs localize to the mitotic spindle, leading to our hypothesis that miR-31 post-transcriptionally regulates its targets at the spindles during mitosis. To test this hypothesis, we also identified EEF1A1 at the mitotic spindle, indicating active translation at the mitotic spindle (Fig 7a)^30^. By using o-propargyl puromycin (OPP), an tRNA mimic that is incorporated into the elongating peptide chain, we observed that newly translated proteins are present throughout the blastomeres, with an enrichment at the mitotic spindle, as well as at the presumptive microtubule organizing center (Fig. 7b). These results indicate that translational machinery is enriched at the spindles and active translation occurs during mitosis.

Since we observe translational machinery, newly translated proteins, and *Fascin* RNA at the mitotic spindle, we hypothesize that *Fascin* is translated at the mitotic spindle. We observed *de novo* translated Fascin protein at the cell periphery and mitotic spindle of dividing sea urchin blastomeres (Fig. 7c). Newly translated Fascin is also observed at the mitotic spindle of dividing HCT116 cells (Fig. 7d), suggesting that active translation of Fascin at the mitotic spindle is evolutionarily conserved.

### miR-31 inhibition results in more Rab35 and Fascin protein at the mitotic spindle.

**Figure 8:**
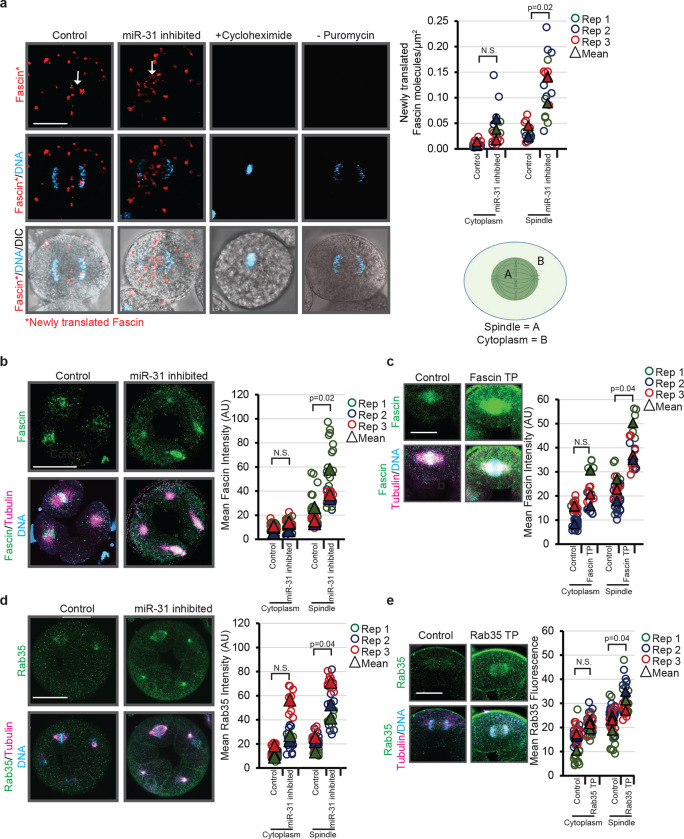
miR-31 inhibition results in increased Fascin and Rab35 protein. (a) Control or miR-31 inhibited 16–32 cell stage embryos were treated with the DuoLink PuroPLA assay with newly translated Fascin in red, and counterstained with DAPI (DNA in blue). The density of newly translated Fascin molecules is significantly higher at the spindle in miR-31 inhibited embryos compared to control embryos. N=16 Control blastomeres, 18 miR-31 inhibited blastomeres. p-value was obtained using Student’s t-test. (b) Embryos were injected with control miR-124 LNA inhibitor or miR-31 LNA inhibitor and immunolabeled with Fascin antibody (green) and tubulin antibody (magenta) followed by DAPI counterstain for DNA (blue). Image J was used to measure the mean fluorescence at the spindle (region A in the schematic) and in the cytoplasm (region B in the schematic). N=29 Control blastomeres, 32 miR-31 inhibited blastomeres. p-value was obtained using Student’s t-test. (b) Embryos were injected with Fascin TP to remove miR-31’s suppression of Fascin. Fascin TP-injected embryos were immunolabeled with Fascin antibody. ImageJ was used to measure the fluorescence intensity of individual blastomeres during anaphase. N=34 Control blastomeres, 23 Fascin TP blastomeres. p-value was obtained using Student’s t-test. Scale bar = 50μm (c) Embryos were injected with control miR-124 LNA inhibitor or miR-31 LNA inhibitor and immunolabeled with Rab35 antibody (green) and tubulin antibody (magenta), followed by DAPI counterstain for DNA (blue). N=39 Control blastomeres, 27 miR-31 inhibited blastomeres. p-value was obtained using Student’s t-test. (d) Embryos were injected with Rab35 TP to remove miR-31’s suppression of Rab35. Rab35 TP-injected embryos were immunolabeled with Rab35 antibody. ImageJ was used to measure the fluorescence intensity of individual blastomeres during anaphase. N=50 Control blastomeres, 31 Rab35 TP blastomeres. p-value was obtained using Student’s t-test. Scale bar = 50μm.

As microRNAs are post-transcriptional regulators that generally negatively mediate gene expression of their target transcripts^16^, we tested if inhibiting miR-31 in the embryo would affect the protein levels of its targets. Upon miR-31 depletion, we observe a general trend of more newly translated Fascin protein compared to the control embryos (Fig. 8a). Importantly, the newly translated Fascin is significantly increased at the mitotic spindle in miR-31 inhibited embryos, further indicating that miR-31 post-transcriptionally inhibit *Fascin* (Fig. 8a). Using conventional immunolabeling in fixed embryos, we also observe a significant increase of Fascin and Rab35 protein at the mitotic spindle in miR-31 inhibited embryos compared to control embryos (Fig. 8b,d). Further, specifically inhibiting miR-31’s binding to *Fascin* (with Fascin TP) or *Rab35* (with Rab35 TP) also resulted in a significant increase of their protein levels at the mitotic spindle (Fig. 8c,e). Importantly, this localization of increased Fascin and Rab35 proteins at the mitotic spindle corresponds to the localization of miR-31, *Fascin*, and *Rab35* transcripts (Figs. 1,5). These data suggest that miR-31 post-transcriptionally regulates *Fascin* and *Rab35* at the mitotic spindle.

### Local translation of Fascin is important for embryonic development.

**Figure 9:**
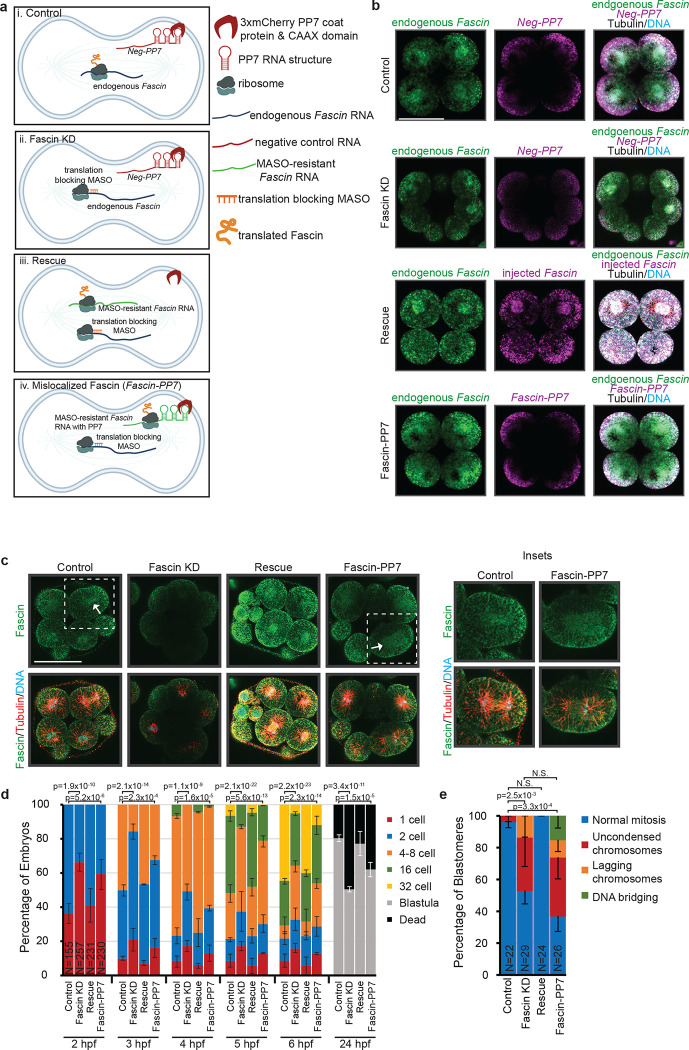
Local translation of Fascin at the mitotic spindle is important for proper cell division. (a) Schematic of components injected for testing importance of local translation of Fascin at the mitotic spindle. All zygotes were injected with the prenylated mCherry PP7 coat protein. (i) Control embryos were also injected with control MASO, and a negative control RNA fused to PP7 repeats (*Neg-PP7*). Endogenous *Fascin* is localized at the spindle and can be translated normally. (ii) Fascin KD embryos were injected with Fascin MASO and *Neg-PP7*. Endogenous *Fascin* transcript is localized at the spindle but cannot be translated due to Fascin MASO binding and inhibiting its translation. (iii) Rescue embryos were injected with Fascin MASO, and Fascin MASO-resistant *Fascin* CDS+3’ UTR. The endogenous *Fascin* localizes to the spindle, but cannot be translated due to Fascin MASO binding, but the exogenous *Fascin* CDS + 3’UTR localizes to the spindle and can be translated. (iv) Fascin-PP7 embryos were injected with Fascin MASO and Fascin MASO-resistant *Fascin* CDS RNA fused to PP7 repeats. Endogenous *Fascin* localizes to the spindle but cannot be translated due to the Fascin MASO, but exogenous *Fascin* localizes to the cell membrane due to the PP7 repeats, forcing translation of Fascin to occur at the cell membrane. (b) Embryos were injected as described in (a). *Fascin* transcripts (green) and exogenously injected transcripts, either *Neg-PP7* or *Fascin-PP7* (magenta), were detected in these embryos. Embryos were also immunolabeled with tubulin antibody (white). (c) Zygotes were injected as described in (a). 16–32 cleavage stage embryos were immunolabeled for Fascin protein (green) and tubulin (red), and counterstained with DAPI for DNA (blue). The insets depict Fascin in individual blastomeres as outlined by the dotted lines. (d) Zygotes were injected with components as depicted in (a). The number of embryos in each stage was recorded every hour for 6 hpf, then again at 24 hpf. No significant difference was observed between the control and rescue groups at all time points. (e) Zygotes were injected as described in (a). Blastomeres undergoing mitosis were scored for chromosomal abnormalities. p-values were obtained using Cochran-Mantel-Haenszel statistical test was used; N.S.= p>0.05. All experiments were conducted in 3 biological replicates. All scale bar = 50μm.

Since our data indicate that miR-31, its target transcripts and proteins have a subcellular localization on the mitotic spindle and that inhibiting miR-31 leads to increased target Fascin and Rab35 protein levels (Figs. 5,8), we tested the importance of local translation of Fascin (Fig. 9a). We tested this by forcing Fascin translation to occur at the cell membrane, in the background of depleted Fascin. The expected result is that if local translation of Fascin was important for mitosis in early cleavage stage embryos, we would observe developmental defects. In this set of experiments, four conditions were tested. In all conditions, the zygotes were injected with the prenylated mCherry-PP7 coat protein which localizes to the cell membrane. To control for injection trauma, the effect of exogenous injection of mRNAs, and morpholino antisense oligonucleotides (MASO) toxicity, we co-injected zygotes with control MASO (against human *β-globin*) and mRNA consisting of a plasmid sequence fused with 3x stem loop structure (referred to as *Neg-PP7* mRNA) that binds to prenylated mCherry-PP7 coat protein at the cell membrane (Fig. 9ai). These control embryos are able to make Fascin protein from endogenous *Fascin* mRNA localized at the spindles (Fig. 9ai,b,c). To deplete Fascin protein, we injected zygotes with Fascin MASO with *Neg-PP7* mRNA (Fig. 9aii). In these embryos, the endogenous *Fascin* mRNA is not translated into Fascin proteins due to MASO binding (Fig. 9aii,b,c), resulting in a significant depletion of Fascin protein. To test the specificity of the Fascin MASO, we co-injected zygotes with Fascin MASO and mRNA consisting of the *Fascin* coding sequence recalcitrant to Fascin MASO binding (lacks Fascin MASO binding sequence) (Fig. 9aiii). As expected, the endogenous and exogenous *Fascin* transcripts are both localized to the spindles (Fig. 9aiii,b). In these Fascin KD embryos, the injected MASO-resistant *Fascin* transcript is translated to protein at the spindles (Fig. 9b,c). These results indicate that Fascin MASO is specifically blocking Fascin translation, as we are able to restore Fascin expression with the exogenous *Fascin* mRNA. To test the importance of local translation of Fascin, we co-injected Fascin MASO with mRNA consisting of the *Fascin* coding sequence recalcitrant to Fascin MASO binding fused to 3x stem loop structure (Fig. 9aiv). In these embryos, the *Fascin* mRNA containing the 3x stem loop structure will bind to prenylated mCherry-PP7 coat protein at the cell membrane; these we refer to as *Fascin-PP7* embryos (Fig. 9aiv). In these Fascin-PP7 embryos, the majority of the endogenous *Fascin* transcript at the spindles fails to be translated due to Fascin MASO, and the embryo’s source of Fascin protein comes from the exogenously injected *Fascin* transcript sequestered at the cell membrane (Fig. 9b). In Fascin-PP7 embryos, we observe that *Fascin* mRNA is enriched at the spindle; however, it has a more diffuse localization compared to the control embryos, potentially due to the *Fascin* RNA probe binding to both endogenous and injected *Fascin* mRNA (Fig. 9b). Interestingly, in these embryos (Fascin-PP7), Fascin protein is localized at the cell membrane, with a wider distribution around the mitotic spindle instead of being enriched at the spindle midzone (Fig. 9c). This indicates that the localization of Fascin protein depends on the subcellular localization of its transcripts.

To test the importance of local translation of Fascin in early development, we followed embryos injected with these four conditions (Fig. 9a) through early development where we tabulated the percentage of embryos at a particular stage for 24 hpf (Fig. 9d). Consistent with our prior study^23^, we observed that Fascin MASO-injected embryos have a significant delay in development as early as 2 hpf, where 60% of control embryos reach 2-cell stage, but less than 40% of Fascin KD embryos have undergone the first division (Fig. 9d). This developmental defect persists to 24 hpf, where 80% and 50% of control and Fascin KD embryos reach the blastula stage, respectively. When endogenous Fascin translation is blocked with a MASO, but exogenous MASO-resistant *Fascin* transcript is supplemented (Fig. 9d), the developmental defect in Fascin KD embryos is rescued. However, intriguingly, Fascin-PP7 embryos with Fascin KD background supplemented with exogenous MASO-resistant *Fascin* transcript that is forced to translate ectopically at the cell membrane, experience a significant delay in development (Fig. 9d). This developmental delay is observed as early as 2 hpf, where 60% of control embryos have reached the 2-cell stage, while only 40% of Fascin-PP7 embryos have divided to 2 cells. Importantly, this developmental difference persists to 24 hpf, where 80% of control embryos have developed to the blastula stage, but only 60% of Fascin-PP7 embryos have reached the blastula stage (Fig. 9d). Additionally, we observe significantly more chromosomal defects in Fascin KD and Fascin-PP7 embryos compared to control and Fascin rescue embryos (Fig. 9e). Overall, results from this set of experiments indicate that the localized translation of Fascin is important for cell division during early embryonic development.

## Discussion

The early cleavage stage of development is a crucial time point that requires exquisite protein regulation, as the embryo undergoes rapid rounds of cell division where rapid regulation of the mitotic proteome is required for proper chromosome segregation^3,18^. We have identified miR-31, an evolutionarily conserved microRNA present in all metazoans^31^, to play an important role in regulating the early cleavage stage sea urchin embryos. miR-31, along with four of its validated targets, *β-actin, Gelsolin, Rab35*, and *Fascin*, have an evolutionarily conserved localization to the mitotic spindle in the sea urchin embryo and mammalian cells (Figs. 1,5,6), suggesting that the regulatory paradigm of miR-31 at the spindles may be conserved.

Prior studies have indicated that a complex pool of RNAs localize to the spindles. For example, we have previously identified that transcripts encoding proteins that regulate mitosis localize to the mitotic spindle^15^, similar to prior studies using *Xenopus* egg extract to identify mitotic spindle-associated transcripts^13,14^. One of these RNA transport mechanisms may depend on the CPEB element. Our prior study indicates that the localization of sea urchin *AuroraB* to the mitotic spindle depends on a putative CPEB element, as is the case of *CyclinB* transcript in several different species, including the fruit fly^32^, purple sea urchin^15^, frog^13^, zebrafish^33^, mouse^33^ and human^11^. Interestingly, our bioinformatics analysis indicates that sea urchin *β-actin, Gelsolin, Rab35*, and *Fascin* all contain putative CPEB elements (UUUUAU or UUUUAAU) in their 3’UTRs. Thus, transport of these transcripts to the mitotic spindle may be mediated by CPEB^11,15^. However, further experiments will be needed to test the mechanism of their transport to the spindles, as other RNA-binding proteins, such as Staufen1 and Vasa, are also known to regulate transport of RNAs to the mitotic spindle^10,12^. While microRNAs have been identified to localize to various subcellular locations, such as the mitochondria^34^ and endosomal pathway compartments^35^, how they are transported to their subcellular localizations is not clear.

The function of miR-31 has been examined in myogenesis, bone homeostasis, skeletogenesis, and in the context of cancer^29,31,36–42^. miR-31 has also been identified to regulate sea urchin skeletogenesis^19,20^. Thus far, miR-31 has not been examined in the context of mitosis or early dividing embryos. Our results indicate that miR-31 perturbation in the sea urchin embryo results in significant developmental delay and lethality with chromosomal defects and ectopically increased cytoskeleton (Fig. 2). Chromosomal defects such as DNA bridging and lagging chromosomes can lead to aneuploidy and cell cycle arrest^43^. In turn, cell cycle arrest could result in the developmental arrest, delay, and death that we observe in miR-31 inhibited embryos (Fig. 2a). Chromosomal defects observed in miR-31 inhibitor-injected embryos may be a result of increased tubulin (Fig. 2). Cytoskeletal dynamics, including levels of tubulin, must be carefully regulated to ensure faithful chromosome segregation during mitosis^44^. When cells overexpress tubulin, defects in chromosome alignment and segregation occur^45^. The exact mechanism of these defects is not clear; however, computational modeling methods have suggested that too many microtubules will prevent proper chromosome congression due to a lack of balanced polar ejection forces^46^. Thus, the increased tubulin we observe in miR-31 inhibited embryos may contribute to the lagging chromosomes and DNA bridges (Fig. 2b,c).

miR-31 inhibition also leads to increased F-actin (Fig. 2d). We found miR-31 directly suppresses *β-actin* (Fig. 3c). This may result in an absolute increase in actin monomers, which would allow more F-actin to be polymerized, either spontaneously or mediated by Arp2/3 or Formin family members^47^. The increase in F-actin in miR-31 inhibited embryos may also be an indirect effect, as miR-31 directly suppresses other transcripts that encode proteins that modulate cytoskeletal dynamics, such as *Fascin*, *Rab35* or *Gelsolin* (Fig. 3c), which in turn may promote formation of F-actin in the embryo. In support of this idea, removal of miR-31’s direct suppression of *Fascin* and *Rab35* results in significant increase of F-actin (Fig. 4c). Since actin plays several other roles during mitosis, increased actin from miR-31 perturbation may negatively impact mitosis. For example, actin polymerization provides forces that separate the newly duplicated centrosomes to opposite sides of the nucleus, just prior to nuclear envelope breakdown^48^. Actin also facilitates initial chromosome congression, by forming a shell with MyosinII around the nucleus which contracts immediately after nuclear envelope breakdown to reduce the chromosomal volume and facilitate spindle microtubules to access the kinetochores^49^. Actin also interacts with microtubules at the cell cortex to mediate spindle orientation^50^ as well as cytokinesis^51^. Additionally, perturbation of actin dynamics results in shorter spindles and prolonged mitosis^5^. Thus, the correct actin dynamics is important for mitosis and increased F-actin we observe in miR-31 inhibited embryos may disrupt various aspects of mitosis (Fig. 2d), potentially contribute to miR-31 inhibitor-induced developmental defects.

Other direct targets of miR-31 that regulate cytoskeletal dynamics including *Gelsolin, Rab35*, and *Fascin* (Fig. 3c). Gelsolin severs and caps actin filaments^52^. While many studies have examined the function of Gelsolin in the context of cancer, inflammation and amyloidosis^52,53^, its function during mitosis remains elusive. *Rab35* encodes a GTPase that is involved in endosomal protein trafficking and regulation of actin dynamics^24,54^. Besides its function in the formation of the cytokinetic furrow^24,25,54^, Rab35’s role during mitosis is limited. We found that removing miR-31’s direct suppression of Rab35 with Rab35 TP results in increased Rab35 protein and chromosomal defects (Fig. 8d,e); however, Rab35 TP results in no change in microtubules or F-actin levels compared to the controls (Fig. 4e,f). Thus, miR-31’s direct suppression of *Rab35* impacts chromosome segregation independent of cytoskeletal changes. Rab35 has been shown to mediate endocytosis of excess plasma membrane in cell shape changes^55^ and cytokinesis^54^. We hypothesize that increased levels of Rab35 protein in Rab35 TP-injected embryos may result in precocious cytokinesis that can lead to unresolved DNA bridges, resulting in DNA damage and embryonic lethality^56^. However, the exact mechanism of how increased Rab35 results in chromosomal segregation defects is unclear.

Prior literature has shown that Rab35 functionally interacts with Fascin^24,57^. The overexpression of Fascin can rescue Rab35 KD phenotypes in bent bristles of fruit flies and gastrulation defects in the sea urchin embryo^24,57^, indicating that Rab35’s functional interaction with Fascin is evolutionarily conserved. Interestingly, we observe both Rab35 and Fascin co-localize to the mitotic spindle with miR-31 and are responsive to miR-31 inhibition, suggesting their potential functional interaction at the spindles. Therefore, Rab35 may regulate mitosis via its interaction with Fascin at the mitotic spindle.

Fascin is an actin bundling protein that also interacts with and directs polymerization of microtubules^21,58,59^. We observed that miR-31 inhibition and removal of miR-31’s direct suppression of *Fascin* resulted in significant increases of Fascin protein, tubulin, and F-actin (Figs. 8b,8c,4b,4c). Consistent with our findings, MDA-MB-231 cancer cells have Fascin overexpression that results in increased microtubule dynamics and is associated with increased metastatic potential^60^. Further, Fascin KD HeLa cells have microtubules that are more stable^21^. Interestingly, we observe *Fascin* transcript to be perinuclear in interphase sea urchin blastomeres and on mitotic spindle in dividing blastomeres and mammalian cells (Figs. 5,6,9b,S3). The level of newly translated Fascin protein at the mitotic spindle is significantly increased in miR-31 inhibited sea urchin embryos (Fig. 8a,b) which can potentially impact cell division in several ways. Nuclear Fascin has been shown to regulate intranuclear actin dynamics^61,62^. Fascin also regulates cancer cell survival by binding to histone H3 to enhance chromatin compaction and recruiting DDR factor _γ_H2AX to damaged DNA foci to facilitate DNA damage response^62^_._ We showed that perturbation of Fascin results in significant chromosomal segregation defects and leads to developmental delay and arrest early in development (Figs. 4a,8,9d,9e). Fascin’s ability and potential to modulate the cytoskeleton in the local environment of the mitotic spindle, regulate intranuclear actin dynamics, and mediate DNA damage repair make it an important protein during mitosis. Further, Fascin may perform an evolutionarily conserved and essential function during mitosis (Figs. 5,6).

As many transcripts that are localized to the mitotic spindle encode for proteins that regulate mitosis^10,11,14,15^, we hypothesize that β-actin, Gelsolin, Rab35, and Fascin may work together by directly mediating actin remodelling at the mitotic spindle, or indirectly, by ensuring that the local microenvironment promotes appropriate microtubule polymerization to ensure proper progression through mitosis^24,57,63^. Our results demonstrated that local translation of Fascin is critical for proper early development, where embryos in which Fascin translation is forced to the cell membrane (Fascin-PP7) do not survive as well as control or Fascin-rescue embryos (Fig. 9d). Interestingly, embryos with mislocalized Fascin have a higher survival rate than Fascin KD embryos. This may be due to the fact that although Fascin translation is forced to occur at the cell membrane, some Fascin can be transported from the cell periphery to the mitotic spindle, or that Fascin KD is not complete. This is supported by our observation that in control embryos, Fascin localizes to the spindles during mitosis; however, in Fascin-PP7 embryos, Fascin protein has a more diffuse localization surrounding the spindle (Fig. 9c inset). While intracellular transport of Fascin has not been well-studied, we have shown previously that kinesin-1 and dynein microtubule motors regulate localization of important mitotic spindle components^15^.

**Figure 10:**
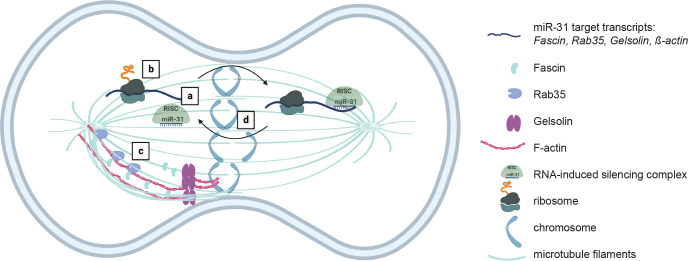
Model of miR-31’s regulation of local translation at the mitotic spindle. a) miR-31 and its target transcripts localize to the mitotic spindle. (b) miR-31 is hypothesized to dynamically regulate translation of its targets during mitosis. (c) miR-31 targets, *β-actin*, *Gelsolin*, *Rab35*, and *Fascin* regulate local cytoskeletal dynamics in order to ensure proper mitotic progression. (d) Defects in this process can lead to chromosome segregation defects.

Evidence suggesting that translation occurs on the mitotic spindle has been around for quite some time, as reviewed by Waldron and Yajima^64^. The first evidence indicated that actively translating ribosomes interacted with the mitotic apparatus, and later, specific mRNAs that interact with the mitotic spindle were identified^14,65^. *CyclinB* has been suggested to be locally translated at the mitotic spindle^13^, as has Vasa^12^. When *CyclinB* transcript is not localized to the mitotic spindle, *Xenopus* embryos have mitotic spindle defects, and are developmentally arrested^13^. When Vasa, an RNA-binding protein, is ectopically forced to the cell membrane, translation is observed to be enriched at the cell periphery and results in failure of the sea urchin embryos to develop micromeres or gastrulate normally^12^. As mitosis requires numerous proteins function together at the correct space and time within the cell, in order to faithfully segregate daughter chromosomes^66^, regulation of local translation by miR-31 at the mitotic spindle may be another mechanism through which the embryo mediate efficient protein translation to regulate spindle dynamics. A failure to control local translation of Fascin at the right place at the right time results in severe chromosomal defects, as demonstrated by the Fascin-PP7 embryos with 62.5% of chromosomal defects compared to the control with 11% of chromosomal defects. Our working model is that miR-31 and its target transcripts localize to the mitotic spindle, where miR-31 dynamically regulates translation of its targets during mitosis (Fig. 10). In post-embryonic cells and cell lines, microRNAs typically inhibit translation of their targets through recruitment of deadenylases to destabilize their target transcripts^16^. However, in pre-blastula stage zebrafish embryos, the regulatory landscape favors translational inhibition as the dominant mechanism^67^. This suggests that during early cleavage stages, miR-31 may interact with its targets dynamically, and without degrading the target mRNAs. We propose that miR-31 regulates *β-actin*, *Gelsolin, Rab35,* and *Fascin* to mediate actin dynamics and microtubule polymerization in order to ensure precise chromosomal segregation and a timely progression through mitosis. Disruption of this process would lead to mitotic arrest, ultimately leading to embryonic lethality (Figs. 9,10). We demonstrate for the first time, that miR-31 regulates *de novo* translation of its targets at the mitotic spindle and a disruption of this local regulation has a negative consequence on early development (Figs. 8,9). Overall, our results highlight the importance of microRNA-mediated post-transcriptional regulation at the spindles of the rapidly dividing early embryo, when efficient protein regulation is critical, and indicate that this regulatory paradigm of mitosis may be evolutionarily conserved.

## Methods

### Animals

Adult *Strongylocentrotus purpuratus* were collected from the California coast (Pt. Loma Marine Invertebrate Lab or Marinus Scientific, LLC.). All animals and cultures were incubated at 12°C.

### Microinjections

Microinjections were performed as previously described with modifications. *Hsa*-miR-31-3p Locked Nucleic Acid (LNA) power inhibitor (Qiagen, Germantown, MD) was resuspended with RNase-free water to 100 μM. All sequences are listed in Table 1. Embryos were injected with 25 μM miR-31 LNA inhibitor, miRCURY LNA miRNA inhibitor negative control A, or 25 μM control miR-124 LNA inhibitor and collected at the 16–32 cell stage to phenotype for developmental defects. To test the importance of local translation of Fascin, we designed a translational blocking MASO (GeneTools, Philomath, OR) against *Fascin*. Fascin translational-blocking MASO, Fascin TP and control MASO (human *β-globin*) were resuspended with RNAse-free water to a 5 mM stock solution and diluted to 2 mM to perform microinjections. Rab35 TP was diluted to 1 mM for microinjections.

Injection solutions contained 20% sterile glycerol, 2 mg/mL 10,000 MW FITC lysine charged dextran (ThermoFisher Scientific, Waltham, MA), and miR-31 LNA power inhibitor, miR-124 LNA power inhibitor, control MASO, or *Fascin* translational-blocking MASO. Injections were performed using the Pneumatic PicoPump with a vacuum (World Precision Instruments; Sarasota, FL). A vertical needle puller PL-10 (Narishige International, USA, INC., Amityville, NY) was used to pull the injection needles (1 mm glass capillaries with filaments) (World Precision Instruments; Sarasota, FL).

### Biotinylated pull down with RNA seq

Embryos were injected with biotinylated miR-31 mimic to identify potential targets^1^. Sequence data for 15 samples (Illumina 50bp single-end) was analyzed by the Center for Bioinformatics and Computational Biology Core Facility at the University of Delaware using the established RNA-Seq analysis pipeline. ^2^. Quality of sequencing data was assessed using FastQCv0.10.1^3^. Reads were trimmed for quality (Q<30) and to remove poly-A and Illumina sequencing adapters using Trim Galore! v0.4.4^4^ with cutadapt v1.13^5^ and reads less than 35 bp after trimming were discarded. Trimmed reads were aligned to the *Strongylocentrotus purpuratus* genome (v3.1 with annotation build 8) housed in the Echinobase^6^ using TopHat2 v2.1.0^7^, mapping metrics were assessed using RseQC v2.6.1^8^, and gene/exon feature counts were calculated using HTseq v0.9.0^9^. Differential expression analysis was performed to identify gene-level features (CPM>1 in two or more samples) which were significantly up or down-regulated between treatment using a generalized linear model accounting for sample-to-sample variation in EdgeR v3.16.5^10,11^ using the likelihood ratio test with FDR correction (q<0.05). To supplement gene annotation information and provide additional context for biological interpretation (e.g. Gene Ontology, Pathway, String network ID, literature PMI, and sequence feature binding sites and modifications), Echinobase gene ID (SPU ID) were mapped to UniProt^12^ entries (release 2017_07): 11,022 (of 11,116) SPU ID mapped to 11,005 UniProtKB protein entries. To identify potential miR-31 binding sites in differentially expressed genes, BLAT^13^ was used to identify potential miR-31 seed sequence (“TCTTGCC”) matches within protein coding genes allowing 1 mismatch.

### Proteomics experiment

Two samples with four biological replicates were studied over two four-plex iTRAQ experiments. Proteomic sample preparation and data collection, starting with lysis (without calcium carbonate) through MSMS data processing, was performed as previously described^14^. Briefly, each sample containing 2000 injected embryos was sonicated and centrifuged. Supernatants were retained for protein precipitation. One hundred μg protein for each sample, as determined by BCA protein concentration assay, were subjected to protein reduction, alkylation, and tryptic digestion. Digested samples underwent detergent removal and iTRAQ labeling; iTRAQ labeled peptides were fractionated offline by high pH RP, then analyzed by low pH RP-ESI-MS/MS. Database searches were conducted using Protein Pilot against a locally downloaded Echinobase database^6,15^. ANOVA analysis was performed with JMP software.

### RNA in situ hybridization

The steps performed for fluorescence RNA *in situ* hybridization (FISH) are described previously with modifications^16^. The *Hsa*-miR-31-3p miRCURY LNA detection probe (Qiagen, Germantown, MD) was used to visualize sea urchin miR-31 (at 0.5 ng/μL) in hybridization buffer and incubated at 50°C for five days. The scrambled-miR miRCURY LNA detection probe was used as a negative control (Qiagen, Germantown, MD, cat# YD00699004) at the same concentration as miR-31 miRCURY LNA RNA probe.

To generate RNA probes against protein-coding transcripts for either the sea urchin or mammalian cell line FISH, PCR was used to amplify transcripts of interest from either sea urchin egg and embryonic cDNA of 24 hpf, 48 hpf, 72 hpf or LLC-PK cells. PCR primers used to make antisense probes are listed in Table 2.

For double FISH *β-actin*, *Fascin, Rab35,* and *Gelsolin* (DNP-labeled, MilliporeSigma, St. Louis, MO) probes were co-incubated with miR-31 (digoxigenin (DIG)-labeled, Roche, Basel, Switzerland). The scrambled miR miRCURY LNA detection probe was used as a negative control with miR-31 FISH. DNP-labeled Firefly was used as the negative control for the DNP-labeled mRNA probe. All RNA probes were added at 0.5 ng/μL. After 4–5 days of hybridization, embryos were incubated with the anti-DIG-POD antibody first overnight at 1:1000 concentration at 4°C and amplified with fluorescein-tyramide using tyramide signal amplification (TSA). The reaction was quenched with 3% hydrogen peroxide in MOPS buffer for 1 h at room temperature (RT). Embryos were incubated with anti-DNP-HRP antibody at 1:250 concentration overnight at 4°C and labeled with Cy5.5 tyramide using TSA (Akoya Biosciences, Marlborough, MA). For single FISH, embryos were incubated in (DIG)-POD antibody overnight at 4°C and labeled with fluorescein-tyramide using tyramide signal amplification (TSA) (Akoya Biosciences, Marlborough, MA).

All FISH embryos were mounted in MOPS buffer and NucBlue (Thermo Fisher Scientific, Waltham, MA). Images were taken using the Zeiss LSM 880 scanning confocal microscope (Carl Zeiss Incorporation, White Plains, NY) (Zeiss LSM 880 with Airyscan Confocal Laser Scanning Microscope (RRID:SCR_020925). The maximum intensity projections of Z-stack images were acquired with Zen software and processed with Adobe Photoshop and Adobe Illustrator (Adobe, San Jose, CA).

LLC-PK^17^ or HCT116^18^ cells were fixed (100 μM MOPS, 0.1% Tween, 4% paraformaldehyde in PBS) for 15 mins at room temperature, then washed with PBS 0.01%Tween. Cells were hybridized as described (Martín-Durán *et al.*, 2017), and incubated with 0.5 ng/μL DNP-labeled *HsFascin* probe and 0.1 ng/ μL *Hsa*-miR-31-3p miRCURY LNA detection probe (Qiagen, Germantown, MD) at 50°C for 48 h. The cells were incubated with anti-digoxigenin-POD antibody at 1:1,000 (MilliporeSigma, Burlington, MA) for 1 h at room temperature and amplified with Tyramide Amplification working solution (1:50 dilution of fluorescein stock with 1x Plus Amplification Diluent-fluorescence) (Akoya Biosciences, Marlborough, MA). Cells were then quenched in 3% hydrogen peroxide for 1 h, followed by incubation in anti-DNP-HRP antibody (Akoya Biosciences, Marlborough, MA) at 1:250 for 1 h, and amplified with Tyramide Amplification working solution (1:50 dilution of Cy5.5 stock with 1x Plus Amplification Diluent-fluorescence) (Akoya Biosciences, Marlborough, MA). Cells were mounted in VectaShield anti-Fade mounting media with DAPI (Vector Laboratories, Newark, CA). Images were obtained with a Zeiss LSM 780 or 880 scanning confocal microscope (Carl Zeiss Incorporation, Thorwood, NY). Single digital image or the maximum intensity projections of Z-stack of images were acquired with Zen software and exported into Adobe Photoshop and Illustrator (Adobe, San Jose, CA) for further processing.

### Immunolabeling procedures

To observe spatial localization of various proteins, immunolabeling was performed using antibodies against various cytoskeletal elements. E7 (anti-β-tubulin) (Developmental Studies Hybridoma Bank, Iowa City, IA, Cat# E7, Lot#2/13/20-54 μg/mL) or anti-α-tubulin (Cat#11224-1-AP, ProteinTech Group, Inc, Rosemont, IL) was used to label microtubules. Anti-Fascin (55K12, ECM Biosciences LLC, Versailles, KY), anti-Rab35 (ProteinTech Group, Inc, Rosemont, IL) and anti-EEF1A1 (Proteintech 11402-1-AP) were used to label Fascin, Rab35 and EEF1A1 respectively. Embryos were fixed in 100% ice-cold methanol for 10 mins on ice for immunolabeling for Fascin, 10% TCA in dH_2_O for 30 mins for immunolabeling for Rab35. Three 15-min Phosphate Buffered Saline 0.1%Triton (PBST) (10X PBS; Bio-Rad, Hercules, CA) washes were performed. Embryos were blocked with 4% sheep serum (MilliporeSigma, St. Louis, MO) for 1 h at RT. Primary antibody incubation was performed with E7 at 1:10, Fascin and EEFA1 at 1:100, and Rab35 at 1:50, respectively. Embryos were washed three times for 15 min with PBST followed by incubation with secondary antibodies goat anti-mouse (for E7 and Fascin) and goat anti-rabbit (α-tubulin and Rab35) Alexa 488 or Alexa 647 at 1:300 for 1 h at RT (Thermo Fisher Scientific, Waltham, MA), sequentially.

To examine F-actin, embryos were labeled with fluorescently conjugated phalloidin as previously described (Konrad and Song, 2022) with minor modifications. AlexaFluor-647 conjugated phalloidin (Thermo Fisher Scientific, Waltham, MA) was reconstituted in DMSO, then diluted to 10U mL^−1^ in PBST. Embryos were washed three times with PBST.

All immunolabeled embryos were counterstained with DAPI in PBST buffer (NucBlue; Thermo Fisher Scientific, Waltham, MA). All immunolabeled embryos were imaged using a Zeiss LSM 880 scanning confocal microscope (Carl Zeiss Incorporation, White Plains, NY) using Zen software (Carl Zeiss Incorporation, White Plains, NY). The maximum intensity projections of Z-stack images were acquired with Zen software (Carl Zeiss Incorporation, White Plains, NY) and processed with Adobe Photoshop and Adobe Illustrator (Adobe, San Jose, CA).

OPP (ThermoFisher Scientific, Waltham, MA) was used as previously described^19^, with minor modifications and the addition of tubulin immunolabeling. Briefly, embryos were incubated with OPP for 30 mins, fixed with 4% paraformaledhyde in ASW. After incubating with the Click-iT cocktail, they were blocked in 4% sheep serum in PBST for 1 h, then incubated overnight with E7 (anti-β-tubulin antibody) at 4°C. Embryos were then incubated in goat anti-mouse Alexa 488 (Thermo Fisher Scientific, Waltham, MA) at 1:300 for 1 h, counter-stained with NucBlue and imaged using a Zeiss LSM 880 scanning confocal microscope (Carl Zeiss Incorporation, White Plains, NY) using Zen software (Carl Zeiss Incorporation, White Plains, NY).

### Cloning of constructs for luciferase assays

The 3’UTR’s of *Fascin* and *Gelsolin,* and the CDS and 3’UTR of *Rab35* and *β-actin* were cloned using sea urchin cDNA into pCR-Blunt vector (Table 2) (Thermo Fisher Scientific, Waltham, MA). Plasmids containing potential cloned DNA inserts were subjected to DNA sequencing (Genewiz Services, South Plainfield, NJ). These were subcloned downstream of the *Renilla* luciferase (*RLuc)* as described previously. The miR-31 binding sites within *Fascin, Rab35, Gelsolin* and *β-actin* were mutagenized at the third and fifth binding sites by using the QuikChange Lightning Kit (Agilent Technologies, San Jose, CA). The sequence of the mutagenesis primers used is listed in Table 2. Clones were sequenced to check for the mutated miR-31 binding site (Genewiz Services, South Plainfield, NJ). Firefly construct (*FF)* was linearized using *SpeI* and *in vitro* transcribed with SP6 RNA polymerase. Transcripts were purified using the RNA Nucleospin Clean-up kit (Macherey-Nagel, Bethlehem, PA). *FF* and reporter *RLuc* constructs were co-injected at 50 ng/μL. 50 embryos at the mesenchyme blastula stage (24 hpf) were collected in 25 μL of 1X Promega passive lysis buffer and vortexed at RT. Dual-luciferase assays were performed using the Promega^™^ Dual-Luciferase^™^ Reporter (DLR^™^) Assay Systems with the Promega^™^ GloMax^™^ 20/20 Luminometry System (Promega, Madison, WI). The rest of the assay was performed as previously described.

### Puro-PLA assay

To assess the subcellular localization of newly translated Fascin, we used the Puro-PLA assay using the DuoLink PLA kit (MilliporeSigma, St. Louis, MO). Experiments in the HCT116 cells were performed as described^20^, with minor modifications. Briefly, cells were incubated overnight on coverslips at 37°C under 5% CO_2_, then incubated for 30 mins in cycloheximide or cell culture media, then incubated in either cycloheximide + 2 μM puromycin, 2 μM puromycin or 0.002% DMSO (solvent negative control). Cells were washed with PBS-MC (PBS, 1mM MgCl_2_, 0.1mM CaCl_2_), then fixed with 4% PFA, 4% sucrose in PBS-MC. Cells were incubated in mouse anti-Fascin antibody (55K12, ECM Biosciences LLC, Versailles, KY) at 1:100 and rabbit anti-puromycin antibody (AbClonal A23031) at 1:1000. Cells were washed and incubated with DuoLink anti-rabbit PLUS and anti-mouse MINUS antibodies at 1:5. After ligation, the DuoLink red detection reagents were used for rolling circle amplification and detection. Cells were washed with PBST three times, then incubated in Alexa-488 conjugated anti-tubulin antibody (Thermo Fisher Scientific, Waltham, MA) (catalog #53-4502-82) overnight. Coverslips were mounted in VectaShield anti-Fade mounting media with DAPI (Vector Laboratories, Newark, CA). Experiments in embryos were performed as described above, with the following modifications: cycloheximide was used at 20 μM, and puromycin was used at 50 μg/mL. Embryos were fixed in 4% PFA in LSW for 1 hour at RT. The puromycin antibody was used at 1:100, and the DuoLink anti-rabbit PLUS and anti-mouse MINUS antibodies were used at 1:5, and embryos were counterstained with NucBlue (Thermo Fisher Scientific, Waltham, MA).

### ImageJ analysis

To quantitatively analyze the change in tubulin, actin, Fascin and Rab35 observed after miR-31 inhibition, single plane confocal images of blastomeres were exported from Zen as TIFFs. Using Image J^21^, a region was drawn as depicted in the figure schematics and the mean fluorescent intensity of the region was measured. These were graphed and a Student’s t-test was used to test for statistical significance. To quantify newly translated Fascin protein after miR-31 inhibition (Fig. 8a), a maximum intensity projection of 5 1 μm thick slices was created in Zen and exported as a TIFF. The number of fluorescent dots, indicative of newly synthesized Fascin, was counted in the mitotic spindle area was and in the cytoplasmic area excluding the mitotic spindle were calculated. The number of dots was then divided by the area to determine the density of newly translated Fascin in each region. The mean density of Fascin in the at the spindle and the cytoplasm for each replicate was calculated, and the means were used to perform a Student’s t-test to calculate the significance between control and miR-31 inhibited embryos.

### Preparation of RNA transcripts for injections

To understand the role that localized translation plays in development, we used the PP7-coat protein and the PP7 RNA motifs previously used to localize and visualize RNA transcripts^22^. We sub-cloned 24 repeats of the PP7 motif (obtained from Addgene#74928) into the pCR-Blunt vector. We cloned the CDS of *Fascin* from cDNA into pCR-Blunt vector (Thermo Fisher Scientific, Waltham, MA), then sub-cloned the Fascin CDS into the PP7 motif containing vector. We also synthesized the Fascin CDS + 3’UTR (Twist Biosciences, San Francisco, CA). These plasmids were linearized with the appropriate restriction enzymes and transcribed using mMessage mMachine kit (Thermo Fisher Scientific, Waltham, MA), and purified using the RNA Nucleospin Clean-up kit (Macherey-Nagel, Bethlehem, PA), and filtered through a Millipore UltraFree 0.22μm filter (MilliporeSigma, St. Louis, MO). Transcripts were injected at 1μg/μL.

### Purification of PP7-mCherry-CAAX

pHR-PP7-2xmCherry-CAAX plasmid (Cat.#74925, Addgene, Watertown, MA) was subcloned into the pNOTAT vector, and transformed into NiCO (DE3) cells (MilliporeSigma, St. Louis, MO). Protein expression was induced with 1mM IPTG (Thermo Fisher Scientific, Waltham, MA) at 30°C. Protein was purified using Ni-NTA Fast Start kit (Qiagen, Germantown, MD), and dialyzed in dialysis buffer (136 mM NaCl, 2.7 mM KCl, 10 mM Na_2_HPO_4_, 1.7 mM NaH_2_PO_4_, 10% glycerol, 0.1 mM DTT, pH 7.0). Protein was aliquoted and stored at −80°C until used for micro-injections.

### Statistical analysis

Statistical tests used were Cochran-Mantel-Haenszel tests, Fisher’s exact or Student’s t-tests. Two-tailed Student’s t-tests were performed in all cases, except when specified as one-tailed. Mean values were calculated for each replicate, then a t-test was performed using the means of each replicate.

## Supplementary Material

Supplement 1

## Data Availability

The data that support the findings of this study are available from the corresponding author upon request.
