# Supplementary material for: miR-31-mediated local translation at the mitotic spindle is important for early development
Source: Res Sq. 2023 Jun 14:rs.3.rs-3044775. Preprint. [Version 1] doi: 10.21203/rs.3.rs-3044775/v1 (PMC10312921; doi:10.21203/rs.3.rs-3044775/v1)
Supplement: Supplement 1 [file NIHPPRS3044775V1-supplement-1.pdf]

Figure S1

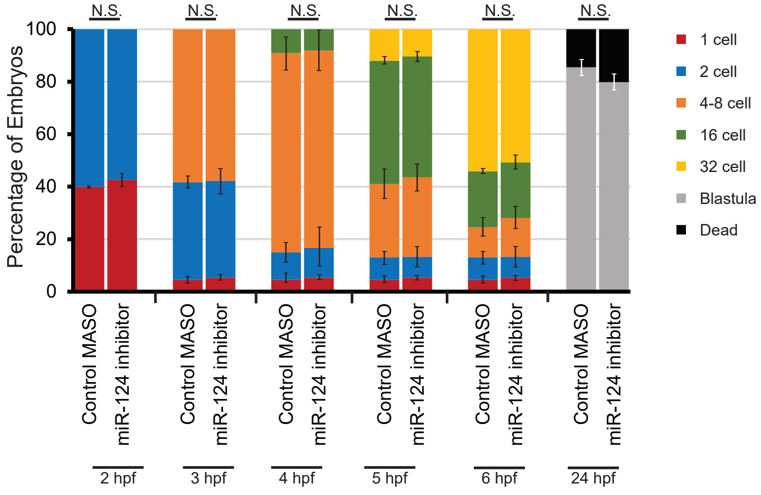

Supplemental Figure 1: miR-124 inhibited embryos do not exhibit a developmental delay when compared to FITC control injected embryos.
Zygotes were injected with control FITC injection solution or control miR-124 LNA inhibitor. The number of embryos in each stage was recorded every hour for 6 hours post-fertilization, then again at 24 hpf. N=310 FITC control injected embryos, 317 control miR-124 inhibitor-injected embryos. N.S. = no significance using Cochran-Mantel-Haenszel test.

Figure S2

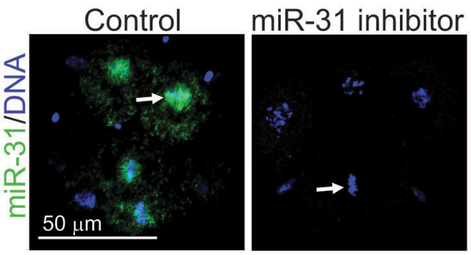

Supplemental Figure 2: miR-31 inhibition leads to decreased levels of miR-31.
(A) Control Texas Red dextran or miR-31 LNA inhibitor were injected into zygotes and cultured to 16-32 cell stage. These embryos were subjected to miR-31 FISH (green) and counterstained with DAPI for DNA (blue). White arrow indicates a dividing blastomere in metaphase.

Figure S3

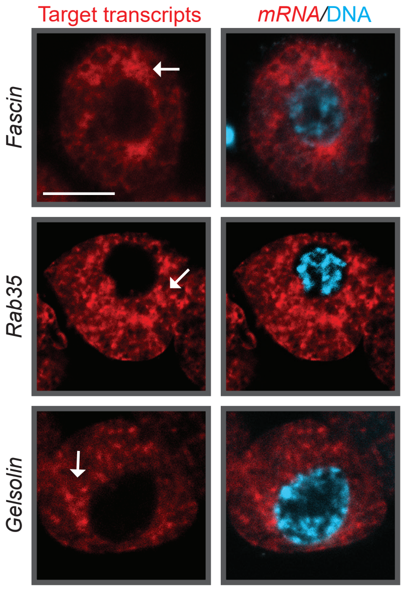

Supplemental Figure 3: miR-31 target transcripts have a perinuclear localization in interphase.
Single blastomeres of a 16-32 cell stage embryo are depicted. 16-32 cleavage stage embryos were subjected to *Fascin, Rab35*, or *Gelsolin* RNA probes (red) and counterstained with DAPI (blue). Arrows indicate the perinuclear localization of miR-31 target transcripts. Scale bar = 10µm. 3 biological replicates.

Table S1. Sequence information

	Sequence (5'-3')
hsa-miR-31-3p miRCURY LNA miRNA Mimic-biotinylated	UGCUAUGCCAACAUAUUGCCAU
miR-31 LNA power inhibitor	AGGCAAGAUGUUGGCAUAGCU
miRCURY LNA miRNA Inhibitor Control	TAACACGTCTATACGCCCA
Control miR-124 LNA power inhibitor	GCATTCACCGCGTGCCTTA
miR-31 LNA *in situ* detection probe	TGCTATGCCAACATATTGCCAT
Scrambled LNA *in situ* detection probe	GTGTAACACGTCTATACGCCCA
Fascin translational MASO	ATCAACATATTTCACAATGCCTGCT
Negative control MASO (Hs β-globin)	CCTCTTACCTCAGTTACAATTTATA
Fascin TP	GAACAGACAAGAGTGCAATGTGACA
Rab35 TP	AGAATGGCAAAAAAACGTAAAGAGT

Table S2. Primer information

	Forward (5' to 3')	Reverse (5' to 3')
**Primers for cloning *in situ* probes** (Sp6 sequence is underlined)		
Sea urchin *Fascin*	GATGGGGATTCGGTAGGTTT	ATTTAGGTGACACTATAGTTCTTAGACGCTGGGACCTG
Sea urchin *Rab35*	ATGGCGAGGGAATACGATCA	TATTTAGGTGACACTATAGTCATTTACCGCAGCATTTTC
Sea urchin *β-actin*	TGTCTTCCCATCTGTTGTCG	ATTTAGGTGACACTATAGTCTTCATGGAGGGTGGAGTC
Sea urchin *Gelsolin*	CGGATCGTCAAGTTCAAGGT	ATTTAGGTGACACTATAGGGCAACCAAGGTGTCTCAGT
Mammalian *Fascin*	GGAGAGCAGGTGGCAATCTT	ATTTAGGTGACACTATAGCCGACCTCCTTTGTTCAGCA
**Primers for cloning into *Renilla luciferase* and mutagenesis (restriction enzyme sites underlined)**		
Fascin	TGCCCTCGAGACAAATTGGGCTTGAAAGAAG	TGCGGCCGCTTTCTAATGACGGCGTGCAT
Fascin seed 1 mut	GTTCAGTGCTTCTGGTATGTAGGAGTTGTCACGGACACTGG	CCAGTGTCCGTGACAACTCCTACATACCAGAAGCACTGAAC
Fascin seed 2 mut	GTATGTTCCATTATCAACAGAACAGATAGGAGTGCAATGTGACAATAAAAAAGTA	TACTTTTTTATTGTCACATTGCACTCCTATCTGTTCTGTTGATAATGGAACATAC
Rab35	CTCGAGTACCGCTCCCTTATGCTAGTGGAC	GCGGCCGCTCTTGTTACAATGACAAAAACAAAAG
Rab35 mut	TCGACACAGAATGGTAGAAAAACGTAAAGAGTATGATAGGGGCG	CGCCCCTATCATACTCTTTACGTTTTTCTACCATTCTGTGTCGA
Gelsolin	GACTGCAGAGCTTTTAGCAACCAAGACGA	GAGGTACCGGGCAACACTGCATTTGGG
Gelsolin mut	CACTGGTCGTTCATGTCAGGTACGACTGATTTTTAAGTGCATG	CATGCACTTAAAAATCAGTCGTACCTGACATGAACGACCAGTG
β-actin	GGTACCATGTGTGACGACGATGTTGC	GCGGCCGCTTAGAAGCACTTCCTGTGGA
β-actin seed 1 mut	ACCGACAACAGATGGTAATACAGCTCGTGGTGCATCG	CGATGCACCACGAGCTGTATTACCATCTGTTGTCGGT
β-actin seed 2 mut	CAAGACGAAGAATGGAATTAGGGAGGGCGTACCCTTCA	TGAAGGGTACGCCCTCCCTAATTCCATTCTTCGTCTTG
β-actin seed 4 mut	GTGGCCGAGGACATACGGTTGGAAGAGGGCTTCGG	CCGAAGCCCTCTTCCAACCGTATGTCCTCGGCCAC

Supplementary Files

This is a list of supplementary files associated with this preprint. Click to download.

- TableS1proteomics.xlsx
- TableS2RNApulldown.xlsx
